# Adjuvant or radical fractionated stereotactic radiotherapy for patients with pituitary functional and nonfunctional macroadenoma

**DOI:** 10.1186/1748-717X-6-169

**Published:** 2011-12-08

**Authors:** Damien C Weber, Shahan Momjian, François P Pralong, Patrick Meyer, Jean Guy Villemure, Alessia Pica

**Affiliations:** 1 Department of Radiation Oncology, Geneva University Hospital, 1211 Geneva, Switzerland; 2 Department of Neurosurgery, Geneva University Hospital, 1211 Geneva, Switzerland; 3 Department of Endocrinology, Centre Hospitalier Universitaire Vaudois (CHUV), 1011 Lausanne, Switzerland; 4 Department of Endocrinology Geneva University Hospital, 1211 Geneva, Switzerland; 5 Department of Neurosurgery, University of Montreal, H3T 1J4 Montreal, Canada; 6 Department of Radiation Oncology, Centre Hospitalier Universitaire Vaudois (CHUV), 1011 Lausanne, Switzerland; 7 Department of Radiation Oncology, Inselspital, 3010 Bern, Switzerland

## Abstract

**Purpose:**

To evaluate the efficacy and toxicity of stereotactic fractionated radiotherapy (SFRT) for patients with pituitary macroadenoma (PMA).

**Methods and Materials:**

Between March 2000 and March 2009, 27 patients (male to female ratio, 1.25) with PMA underwent SFRT (median dose, 50.4 Gy). Mean age of the patients was 56.5 years (range, 20.3 - 77.4). In all but one patient, SFRT was administered for salvage treatment after surgical resection (transphenoidal resection in 23, transphenoidal resection followed by craniotomy in 2 and multiple transphenoidal resections in another patient). In 10 (37%) patients, the PMAs were functional (3 ACTH-secreting, 3 prolactinomas, 2 growth hormone-secreting and 2 multiple hormone-secretion). Three (11.1%) and 9 (33.3%) patients had PMA abutting and compressing the optic chiasm, respectively. Mean tumor volume was 2.9 ± 4.6 cm^3^. Eighteen (66.7%) patients had hypopituitarism prior to SFRT. The mean follow-up period after SFRT was 72.4 ± 37.2 months.

**Results:**

Tumor size decreased for 6 (22.2%) patients and remained unchanged for 19 (70.4%) other patients. Two (7.4%) patients had tumor growth inside the prescribed treatment volume. The estimated 5-year tumor growth control was 95.5% after SFRT. Biochemical remission occurred in 3 (30%) patients with functional PMA. Two patients with normal anterior pituitary function before SFRT developed new deficits 25 and 65 months after treatment. The 5-year survival without new anterior pituitary deficit was thus 95.8%. Five patients with visual field defect had improved visual function and 1 patient with no visual defect prior to SFRT, but an optic chiasm abutting tumor, had a decline in visual function. The estimated 5-year vision and pituitary function preservation rates were 93.2% and 95.8%, respectively.

**Conclusions:**

SFRT is a safe and effective treatment for patients with PMA, although longer follow-up is needed to evaluate long-term outcomes. In this study, approximately 1 patient with visual field defect out of two had an improved visual function.

## Introduction

Pituitary adenomas are usually benign tumors that account for 10 - 15% of primary intracranial neoplasms[1]. Tumor growth frequently causes compression of the visual apparatus, pituitary stalk, floor of the third ventricle and enlargement of the sella turcica[2,3]. Pituitary macroadenoma (PMA) refers to tumors more than 10 mm in diameter, although this cutoff is not consensual[4]. Although debulking surgery is indeed beneficial in a substantial number of PMA patients, long term tumor and endocrinological control remains more often than not elusive, as a result of the tumor extension, superiorly into the suprasellar cistern and/or laterally in the cavernous sinus. Complete resection is achievable in only 44 - 84% of patients with PMA[5]. As such, postoperative[6,7], salvage[8] or radical radiation therapy (RT) is frequently administered to patients with pituitary tumors in general, and to PMA patients in particular. RT has been proven to be an effective treatment in retrospective series for controlling hormone production and tumor growth, with its consequential mass-effect on the adjacent brain structures[9-12].

RT can be delivered by conventional 3D-conformal delivery-techniques or newer radiation techniques, allowing for better target dose-conformation, including but not limited to intensity modulated RT[13], proton beam therapy[14], radiosurgery[15-17] or stereotactic fractionated RT (SFRT)[1,18,19]. In this report, we examined the efficacy and toxicity of SFRT for PMA in 27 patients with long term follow-up treated in an academic center.

## Methods and materials

The institutional database of the departments of Radiation Oncology of Geneva University Hospital (HUG) and Lausanne University Hospital (CHUV) were queried. Eligibility criteria for this retrospective analysis were: 1) PMA; 2) SFRT with definitive intent (patients receiving palliative radiotherapy were not included in this study) and 3) complete endocrinological, radiological and ophthalmological follow-up available. Between March 2000 and March 2009, 27 such patients were identified and underwent SFRT at CHUV, using a linear accelerator dedicated to stereotactic radiation therapy (Siemens USA, New York, NY). PMA patients seen at HUG were referred to the CHUV for SFRT. This retrospective study was approved by both the CHUV's and HUG's Institutional Review Boards. The medical records of these patients, followed by the Endocrinology and Ophthalmology departments of both centers, were reviewed through the most recent follow-up visit with respect to pituitary function, visual function and radiological changes in tumor volume. The acute and late toxicity, possibly related to SFRT, was assessed during routine follow-up in the CHUV and HUG radiation oncology departments.

Prior to SFRT, all patients were evaluated by a multidisciplinary tumor board composed of endocrinologists, neurosurgeons, neuroradiologists and radiation oncologists before and after the initiation of therapy.

The baseline characteristics of the PMA 27 patients (male, *n *= 15; female, *n *= 12) are detailed in Table 1. Median age was 56.5 years (range, 20.3 - 77.4). Of the 27 patients, 26 with newly diagnosed PMA underwent transsphenoidal surgery (TSS) as initial therapy, with or without craniotomy (Table 1). Of these patients, 2 and 1 had TSS followed by craniotomy and TSS followed by a repeated TSS procedure, respectively. One patient with Cushing disease did not have surgery, as the PMA was evaluated to be invasive and thus non-resectable (Table 1) and this patient underwent radical SFRT. Ten (37%; Table 1) patients had functional (ACTH, *n *= 3; prolactin, *n *= 3; GH, *n *= 2; ACTH/GH, *n *= 1 and GH/PRL, *n *= 1) PMA and these patients received antisecretory medication during SFRT. Patients were considered for SFRT if they had visual function, with or without mass effect on the adjacent optic apparatus, progressive radiological and/or biological disease and definable tumor on brain MRI. SFRT was administered 4.2 to 242.6 months after the initial TSS (median, 22.4).

**Table 1:** Patient's and treatment characteristics

	No. of patients (%)
Type of PMA	
Functional	10 (37.0)
Non-functional	17 (63.0)
	
Prior surgery	
None	1 (3.7)
One	23 (85.2)
Two	3 (11.1)
	
Type of surgery	
TSH	
one	23 (85.2)
Two	1 (3.7)
Craniotomy and TSH	2 (7.4)
	
Prior radiotherapy	0 (0.0)
	
Visual field defect	
No	17 (63.0)
Yes	10 (37.0)
	
Visual tract and PMA	
PMA not abutting visual tract	15 (55.6)
PMA abutting visual tract	3 (11.1)
PMA compressing visual tract	9 (33.3)
	
Endocrine function	
Normal	9 (33.3)
Partial anterior pituitary deficiency	18 (66.7)
Complete anterior pituitary deficiency	0 (0.0)
	

PMA, Pituitary macroadenoma; TSH, transsphenoidal hypophyectomy.

A thermoplastic stereotactic mask (BrainLAB, Feldkirchen, Germany) was used for SFRT. Gross tumor volume (GTV) was delineated in the Brainscan (Ver. 5.21) treatment planning system, using image fusion from the CT and MRI datasets. All GTVs and organs at risk (OARs), including but not limited to the optic apparatus, brainstem and temporal lobes, were identified and defined by the same radiation oncologist (AP). The planned target volume (PTV) was defined as the GTV + 2.5 mm. Isodose prescriptions were based on the isodose volume that most closely approximated the PTV. Two third (*n *= 18) of the prescription isodose were 100%. Six and 3 patients were treated at the 85% and 90% isodose line, respectively. The dose constraints to the brainstem, eyeball and temporal lobes were 54, 10 and 30 Gy, respectively. The maximum dose to the optic chiasm and optic nerve was kept below 54 Gy. All dose constraints were met during SFRT planning.

All patients were treated with 1.8 Gy dose per fraction. Median total administered dose was 50.4 Gy (range, 45.0 - 54.0). The use of stereotactic conformal technique provided appropriate coverage of the prescribed dose to the PTV with a median conformity index of 1.2 (range, 1-1.7).

### Follow-up evaluation

After SFRT, patients were followed at 3, 6 and 12 months in the first year and yearly thereafter for endocrine workup. Serial brain imaging studies (MRI) were requested usually at 6 months and 1 year after SFRT, annually for the next 2 years and one once every second or third year thereafter. Neuro-ophtalmologic follow up was performed yearly. Treatment failure was defined as interval growth demonstrated on serial post-SFRT MRI scans and/or increase in hormone production. Acute toxicities were defined as those adverse events that occur from the first day of the treatment through day 90 after treatment. All side effects seen after 90 days from the end of SFRT were considered late complications. Acute and late complications were classified according to the National Cancer Institute Common Terminology Criteria for Adverse Events (CTCAE) v3.0 grading system (http://www.eortc.be/services/doc/ctc/ctcaev3.pdf), except for the skin erythema which was scored using the Radiation Therapy Oncology Group (RTOG) scoring system (http://www.rtog.org/ResearchAssociates/AdverseEventReporting/CooperativeGroupCommonToxicityCriteria.aspx). All patients were followed > 1 year and none were lost to follow-up. The mean follow-up time was 72.4 ± 37.2 months.

### Statistical analysis

Adenoma volumes were measured in accordance with the 2000 guidelines to evaluate tumor response[20]. Three orthogonal diameters were measured from MRI scans taken before and at intervals after FSRT, and tumor volume was calculated. Partial response was defined as tumor shrinkage ≥ 25% and complete response as no visible tumor. Tumor growth control was calculated from the date of SFRT using Kaplan-Meier estimates[21]. The events were local progression or death for treatment failure. In secreting tumors, the secondary endpoint was the normalization of hormonal hypersecretion. Biochemical complete response rate for acromegaly was defined by basal GH levels < 2.5 ng/ml or glucose-suppressed GH levels < 1 ng/ml and normal IGF-I values. Normalization of prolactin hypersecretion was defined as prolactin basal levels in the normal range for men (3 - 16 ng/ml) and women (3 - 23 ng/ml). Normalization of Cushing disease was defined by normal cortisol levels. The actuarial visual preservation and newly pituitary dysfunction were also evaluated using the Kaplan-Meier method[21]. In patients without prior visual compromise, the events for newly visual toxicity after SFRT was objective visual dysfunction scores as the uncensored event. Patients considered at risk of loss of pituitary function after SFRT included patient with either normal pituitary or hypopituitary function. Proportions were compared using the Fisher's exact test for values ≤ 5. All statistical tests were two sided, with alpha levels lower than .05 considered statistically significant. All analyses were performed using the SPSS statistical package (SPSS 17.0, Chicago, IL; http://www.spss.com).

## Results

**Figure 1:**
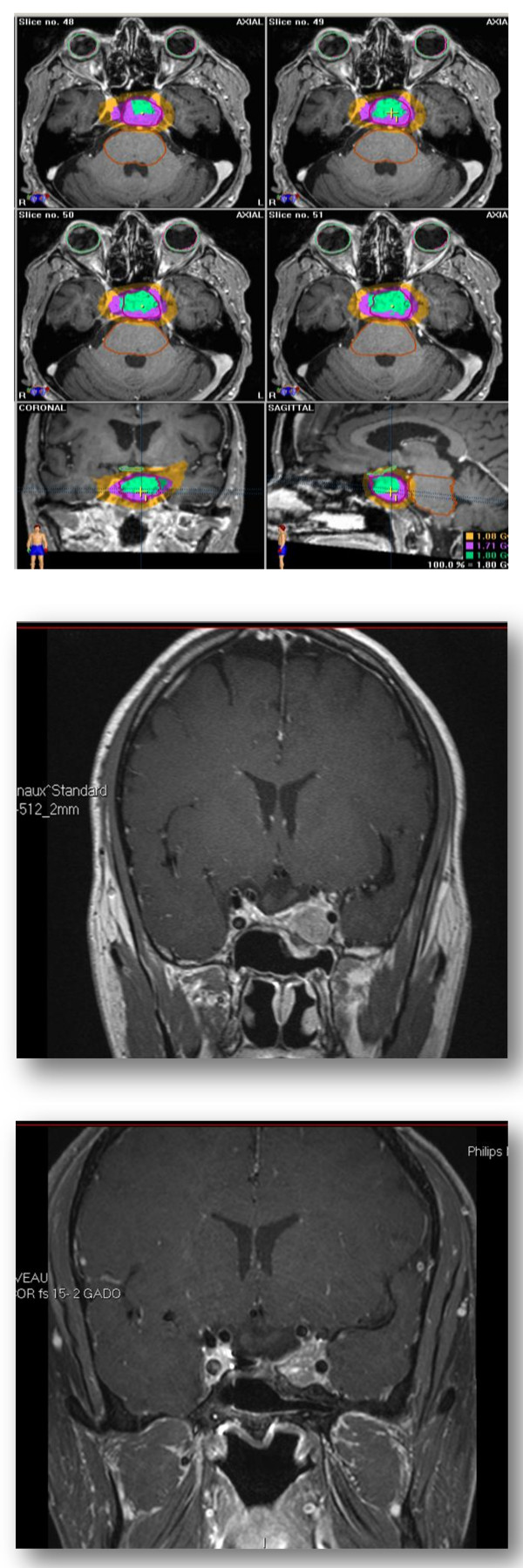
**Pre- and post-treatment MRI and CT scan with the dose deposition**.

All patients were able to complete SFRT. An example of a treatment is displayed in Figure 1. Mean GTV and PTV was 2.9 ± 4.6 cm^3 ^and 8.4 ± 5.8 cm^3^, respectively. The median dose delivered to the optic chiasm and brainstem was 50 Gy (range, 24.5 - 54.0; mean 47.7 ± 7.3) and 41.0 Gy (range, 13.0 - 52.3; mean, 37.2 ± 12.7), respectively. Three patients had treatment interruptions (mean, 4.3 days) for technical reasons pertaining to the Linac. Ten (37%) patients had acute grade 1 - 2 toxicity: 4 patients presented with grade 1 headache, 3 patients with grade 1 - 2 asthenia (grade 2, *n *= 1), 2 patients with grade 1 - 2 nausea (grade 2, *n *= 1) and another patient presented with transient grade 1 visual compromise. No grade > 2 acute toxicity was observed. No cranial nerve dysfunction was observed.

### Outcome and Growth control

**Figure 2:**
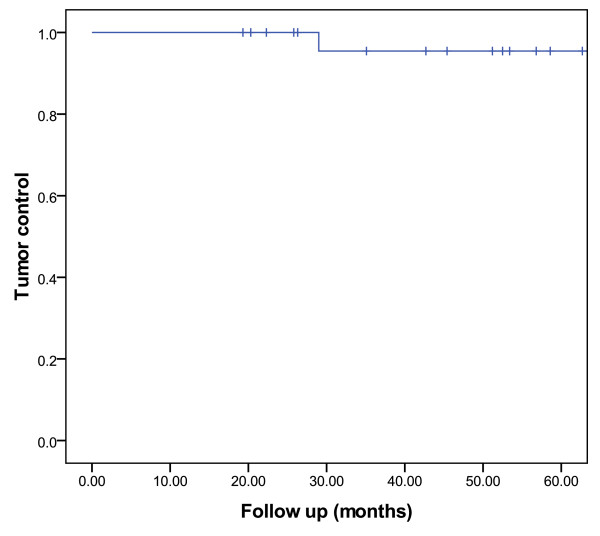
**Estimated tumor growth control**.

At last follow-up, all patients were alive. Twenty five patients presented no radiological tumor progression for non-functional PMA (*n *= 15) and no radiological tumor progression and biological progression for functional PMA in another 10 patients. Two (7.4%) patients with functional (ACTH and GH/PRL) macroadenomas presented with tumor progression, 29.0 and 105.4 months after treatment, respectively. The estimated tumor growth control was 95.5% [_95% _CI: 86.9 - 100.0] at 5 years after SFRT (Figure 2). One patient was salvaged by total hypophysectomy and another underwent Gamma-knife radiosurgery associated with octreotide administration.

MRI scans performed after SFRT demonstrated that the tumor had decrease in size in 6 (22.2%) patients and remained unchanged for 19 (70.4%) other patients. An example of tumor shrinkage is depicted in Figure 1.

### Visual and endocrine outcome

**Figure 3:**
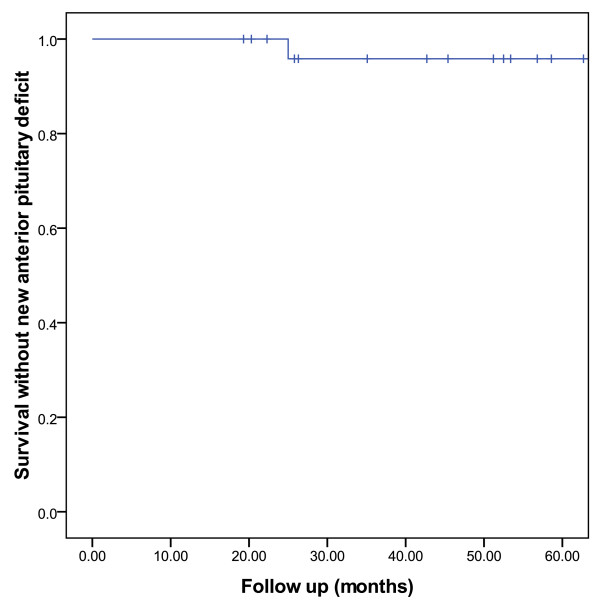
**Estimated rate of anterior pituitary function preservation**.

Before SFRT, patients 18 (66.7%) had decreased pituitary function and 9 (33.3%) had normal function. Of the latter group, 2 (22.2%) patient developed new anterior pituitary deficits. The estimated 5-year survival without new anterior pituitary deficit (Figure 3) was thus 95.8% [_95% _CI: 87.6 - 100.0]. No patient developed panhypopituitarism.

Of the 27 patients, 12 (44.4%) patients had a tumor abutting or compressing the optic apparatus (Table 1) and 10 (37.0%) had an objective visual field defect before SFRT. After this treatment, the visual field had improved in 5 (50%) patients and remained stable in all others. Of note, one patient with no visual dysfunction but an abutting tumor on the optic chiasm prior to SFRT developed a bilateral optic neuropathy 8 months after SFRT, based on the review of formal Humphrey perimetry. This patient received a dose of 50.4 Gy with a Dmax for the chiasm of 49.8 Gy and for the optic nerves of 49 Gy. The actuarial 3- and 5-year vision preservation was thus 95.5% [_95% _CI: 86.9 - 100.0].

**Figure 4:**
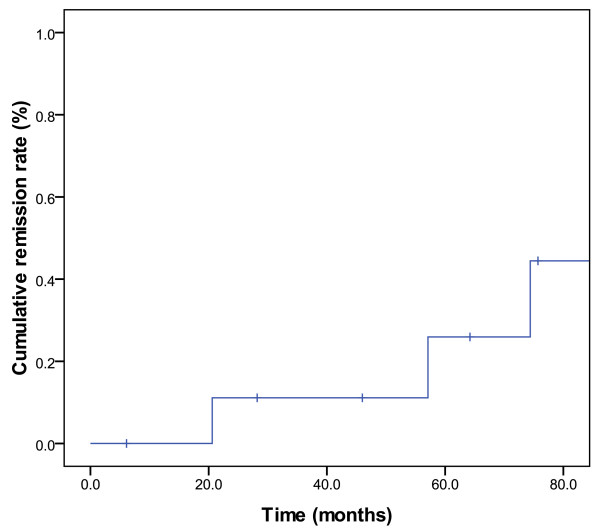
**Cumulative hormone remission rate**.

Serial changes for hormonal levels were evaluated in 10 functional PMA. Of these, true biochemical remission rate occurred in 3 (30%) patients and treatment failed in 7 (70%) patients. One patient with a GH-secreting PMA had no somatostatin analogs and normal levels of IGF-1 five years after SFRT. Biochemical remission was observed in two other patients with prolactin- and ACTH-secreting tumors, respectively. The cumulative hormone remission rate is illustrated in Figure 4. No difference in hormone remission was observed in ACTH-secreting and non-ACTH-secreting PMA was observed (*p *= 0.99).

## Discussion

The results of this study suggest that SFRT achieves high (> 95%) local control (Figure 2) for PMA with exceptional radiation-induced toxicity. The observed tumor control rates compares favorably with other SFRT series for smaller pituitary tumors[18,19,22-24]. Gamma knife-radiosurgery (SRS) has also been administered to patients with PMA [15,25]. Owing to the biological advantage of a single fraction of radiation, an increase in objective radiological response (i.e. 60 - 70%) is usually observed after SRS[16,25] when compared to SFRT. SRS is usually limited however to smaller tumors, in vicinity (≥ 2-3 mm) of the optic apparatus but not abutting this critical structure and the radiosurgery dose constraint for the optic nerve and chiasm is usually 8 Gy. This dose constraint derives the observed 78% risk of optic neuropathy in patients receiving > 15 Gy, when compared to 27% when the radiosurgical delivered dose to the optic apparatus is the range of 10 to 15 Gy[26]. Newly diagnosed pituitary dysfunction after SRS is reported in 0 - 50% of patients with normal pituitary function[7,27-30]. This incidence of radiation-induced toxicity is alleged to be higher than the observed hypopituitarism rate after FSRT[23], although data support that the SRS-SFRT toxicity profile is probably comparable[24]; pituitary dysfunction is likely however to be higher when patients are followed long term, such as those treated with SRS. The indication for SRS thus depends on the target volume and distance to the optic apparatus. If a small adenoma is not located in vicinity of the chiasm, we would recommend SRS. Alternatively, a large tumor in immediate proximity to the chiasm should be treated with SFRT.

In our series, we have observed an absolute improvement of visual field defect and 5-year vision preservation rate of 50% and 93.3%, respectively. Noteworthy, approximately one patient out of two had either a tumor abutting or compressing the optic apparatus (Table 1). These results may be in keeping with other SFRT series which reported an improvement of field defect and vision preservation rate of 0 - 40%[1,18] and 93 - 100%[18,22,23], respectively. Importantly, the choice of keeping the dose per fraction < 2 Gy in all patients was appropriate, on the basis of the data from Parsons *et al*[31]. The tolerance of the optic apparatus might be lower in patients with pituitary adenomas, as visual toxicity has been observed at doses as low as 46 Gy[13,32,33]. Using SFRT for dose escalation, the Thomas Jefferson group has observed two cases with visual loss at 50 Gy delivered with conventional fractionation and has consequently decreased the dose per fraction at 1.8 Gy[18].

Pituitary hormone deficiency is a frequent (30 - 50%) complication after conventional postoperative RT for PMA[7]. In our series, a limited number of patients presented new anterior pituitary deficits after SFRT and the estimated 5-year survival without new anterior pituitary deficits is 96% (Figure 3). These results are comparable to those reported by other SFRT series with smaller pituitary tumors[18,22,23,34-36]. We have also observed a 30% hormonal response rate in the 10 patients with functional PMAs. This observed response rate is in line with other series of SFRT for pituitary tumors[19,22,36,37]. Interestingly, one French prospective study reported a hormonal superior response rate when treated with SFRT only (62%), when compared to SFRT and surgery (42%)[19]. Conversely, in a retrospective comparison of radiosurgery and 3D-CRT, the 2- and 4-year hormonal complete remission rate was regardless of the treatment modality 26.2% and 76.3%, respectively[38]. The median time to complete remission was however significantly (*p *= 0.007) longer in the non-radiosurgery group (63 months) when compared to the radiosurgery group (26 months)[38]. The prolonged time to hormone normalization was also observed with SFRT (median, 18 months) when compared to radiosurgery (median, 8.5 months) in another series[22].

With a mean follow-up period of 6 years, we have not observed any brain necrosis, cerebrovascular disease or radiation-induced tumors. During the carcinogenesis period for the latter complication, there is a latency time between exposure to radiation and cancer onset of many decades. A strong disclaimer should thus be made on the absence of any observed radiation-induced tumors in our series. The patient must always be informed of this dire complication, as the estimated 10 - 30 years cumulative actuarial risk of developing an in-field secondary cancer is 2 - 3%[12,39,40]. Although we did not assess specifically the cognitive function of our patients, no dementia or major cognitive impairment was observed during the follow-up period. The effect of radiation on patient's cognitive function is however controversial, as patient with pituitary adenoma have suboptimal cognition (anterograde memory[41], verbal memory and executive functioning deficits[42,43]) when compared to normal controls. A recent Dutch study compared the cognitive function, using validated cognitive tests, of adult patients with nonfunctioning PMA who underwent transsphenoidal surgery, with or without RT. Patients treated with radiation did not show significant cognition scores when compared to those treated with surgery only, although these results are somewhat controversial[44].

There were several limitations of our study. First, the study was retrospective in nature and thus lacked data for certain important variables such as patient's neurocognitive function and Quality of Life. The small sample size of 27 patients limits somehow the generalizability of these results. This being said, the patient cohort studied was unselected, treated with the same physicians and the follow-up period, extending to over 10 years, is substantial.

In summary, SFRT achieves effective tumor control in patients with PMA with a low incidence of visual or endocrinological impairment. No cranial nerve dysfunction or radiation-induced tumors was observed at follow-up.

## Abbreviations

PMA: Pituitary macroadenoma; RT: radiation therapy; SFRT: stereotactic fractionated RT; HUG: Geneva University Hospital; CHUV: Lausanne University Hospital; TSS: Transsphenoidal surgery; GTV: Gross tumor volume; OAR: organ at risk; PTV: Planned target volume; MRI: magnetic resonance imagery; CTCAE: National Cancer Institute Common Terminology Criteria for Adverse Events; RTOG: Radiation Therapy Oncology Group.

## Competing interests

The authors declare that they have no competing interests.

## Authors' contributions

DCW and AP were responsible for the primary concept and the design of the study; DCW, FPP, PM and AP, performed the data capture and analysis. DCW drafted the manuscript; DCW performed the statistical analysis; DCW and AP reviewed patient data; all authors revised the manuscript. All authors have read and approved the final manuscript.
